# An X-Band Radar System for Bathymetry and Wave Field Analysis in a Harbour Area

**DOI:** 10.3390/s150101691

**Published:** 2015-01-14

**Authors:** Giovanni Ludeno, Ferdinando Reale, Fabio Dentale, Eugenio Pugliese Carratelli, Antonio Natale, Francesco Soldovieri, Francesco Serafino

**Affiliations:** 1 Institute for Electromagnetic Sensing of Environment (IREA), Italian National Research Council (CNR), Via Diocleziano 328, 80124 Napoli, Italy; E-Mails: ludeno.g@irea.cnr.it (G.L.); natale.a@irea.cnr.it (A.N.); soldovieri.f@irea.cnr.it (F.S.); serafino.f@irea.cnr.it (F.S.); 2 Department of the Industrial and Information Engineering, Second University of Naples, Via Roma 29, 81031 Aversa (CE), Italy; 3 Maritime Engineering Division University of Salerno (MEDUS), Department of Civil Engineering of University of Salerno, Via Giovanni Paolo II 132, 84084 Fisciano (SA), Italy; E-Mails: freale@unisa.it (F.R.); epc@unisa.it (E.P.C.)

**Keywords:** marine X-band radar, sea state monitoring, radar bathymetry, sea wave field, reflected sea waves

## Abstract

Marine X-band radar based systems are well tested to provide information about sea state and bathymetry. It is also well known that complex geometries and non-uniform bathymetries provide a much bigger challenge than offshore scenarios. In order to tackle this issue a retrieval method is proposed, based on spatial partitioning of the data and the application of the Normalized Scalar Product (NSP), which is an innovative procedure for the joint estimation of bathymetry and surface currents. The strategy is then applied to radar data acquired around a harbour entrance, and results show that the reconstructed bathymetry compares well with ground truth data obtained by an echo-sounder campaign, thus proving the reliability of the whole procedure. The spectrum thus retrieved is then analysed to show the evidence of reflected waves from the harbour jetties, as confirmed by chain of hydrodynamic models of the sea wave field. The possibility of using a land based radar to reveal sea wave reflection is entirely new and may open up new operational applications of the system.

## 1. Introduction

As it is well known, X-band radar can be used to extract valuable information about the sea state; the main mechanism is the interaction between the electromagnetic waves and the sea's short capillary waves, which in turn ride over longer gravity waves, and has been described and tested for many incidence angles and wavelengths (see for instance [1–6]). In the last two decades various approaches have been developed and validated to estimate sea state parameters such as the Significant Wave Height (SWH), the wave spectrum, as well as surface currents and bathymetry [7–10].

Most of the systems employed to these purposes operate in the short pulse mode (*i.e*., pulse duration of about 50 ns) and are equipped with a 9-ft (about 2.74 m) antenna. These features enable them to attain a range resolution of about 7 m and an angular resolution of approximately 0.9° thus providing good results in sea state monitoring [10–13]. Cheaper devices (*i.e.*, 4 or 6-ft antenna radar) have also been applied but usually, due to their poor angular resolution, they cannot provide adequate results, above all in a low signal to noise ratio (SNR) regime [14].

It is also well known that the non-uniform bathymetry and current fields typical of coastal areas can complicate the estimation of the hydrodynamic parameters (*i.e.*, the direction, the period and the wavelength of the dominant waves) with respect to offshore situations [10].

The present paper is aimed at describing an application of the Remocean processing system—implemented and tested at the Institute for Electromagnetic Sensing of the Environment (IREA) of the Italian National Research Council (CNR)—to provide reliable estimates of the surface currents, bathymetry and hydrodynamic parameters in such challenging conditions [15], by making use of an ordinary X-band navigation radar on a commercial ship during a brief stopover in a harbour.

The method proposed here involves the spatial partitioning of the radar data [16,17] and makes use of the Normalized Scalar Product (NSP) strategy to extract the bathymetry and surface current maps [7].

A comparison is provided between the reconstructed bathymetry field and the nautical chart of the area, in order to confirm the reliability of the NSP method in the nearshore zone; this implies that the filtering of the image spectrum by making use of the bathymetry is accurate. By analysing the reconstructed wave spectrum, a definite evidence was found of reflected waves: in order to confirm this result, and since no wave meters were available at or near the test site, a reliable estimate of the sea state was obtained through a chain of numerical models, transforming the offshore SWH values provided by the European Centre for Medium-Range Weather Forecast (ECMWF) into the nearshore wave field through the well-known Simulating Waves Nearshore (SWAN) software [18,19]. A refraction/reflection near-field model was then used to understand near-field effects and in particular to highlight the presence of reflected waves. This is perhaps the most important result of the work reported here, and it might potentially evolve into an important feature of X-band radar sea monitoring systems, since reflected waves may significantly complicate the harbour activities (e.g., berthing operations), as they interfere with the oncoming waves thus creating a confused sea [20,21].

The paper is organized as follows: in Section 2 the data processing approach to estimate the surface currents, the bathymetry and the sea state parameters is briefly discussed. Section 3 describes both the test area and the hardware used to acquire the images. In Section 4 we deal with the validation of the reconstructed bathymetry field by echo-sounder measurements of the depth. In Section 5, the detection of reflected waves is discussed.

## 2. Data Processing for Sea State Monitoring in a Coastal Area

The strategy employed to retrieve the sea state from nautical radar data typically involves the processing of a temporal sequence of *N_t_* partially overlapping consecutive radar images. Starting from the 3D spectrum of the radar sequence and by going through a number of operations to take into account the physical processes and to filter out the distortions [22], it is possible to extract the wave spectrum and the hydrodynamic parameters, as well as the surface currents and the bathymetry.

The commonly used procedure is based on the assumption that the former quantities do not vary significantly in the scene; this assumption is normally only true for deep offshore areas, where the physical parameters can be reasonably assumed to be spatially homogeneous [11–13]. In many instances such as in the case considered here, the data do not meet this requirement due to the non-uniform surface currents and bathymetry fields; the retrieval of the sea parameters requires therefore a local estimation procedure. Accordingly, a strategy has been used based on the spatial partitioning of the radar images into partially overlapping patches [12,13,16,17].

**Figure 1.**
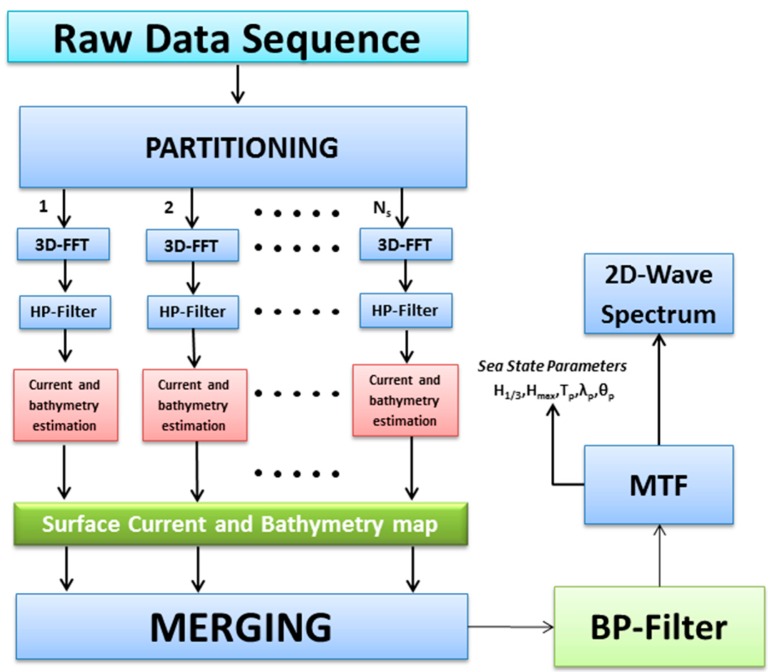
Block diagram of the reconstruction procedure.

The size of such patches has to be found as a trade-off between the minimum number of samples for the spectral analysis and the maximum extension of the area that may be considered homogeneous. The block diagram of this data processing procedure is summarized in Figure 1.

Each radar image within the temporal sequence is partitioned into *N_s_* uniform spatial patches, thus producing *N_s_* temporal sub-sequences and—by using a FFT (Fast Fourier Transform) algorithm—*N_s_* 3D spectra. Each spectrum is then high-pass (HP) filtered to compensate the power decay which affects the radar signal along the range direction. We denote this set of filtered signals with ${\left\{F_{I}^{j}\left(\bar{k},\omega \right)\right\}}_{j=1,\ldots ,N_{S}},\bar{k}=\left(k_{x},k_{y}\right)$ being the wave-vector and ω the angular frequency. The local joint estimation of the bathymetry and surface current is then carried out by applying the NSP algorithm to each spectrum of the set. The NSP algorithm—like most similar systems—exploits the dispersion relation of the gravity waves to extract the sea signal from the overall noisy data [7]. The analytic expression of the dispersion relation for the sea gravity waves is given by Equation (1):

(1) $$\omega \left(\bar{k}\right)=\sqrt{gk\tanh\left(kh\right)}+\bar{k}\times Ū$$

where *g* is the gravity acceleration, *Ū* = (*U_x_,U_y_*) is vector of the sea surface current, *h* is the depth and $k=\left|\bar{k}\right|=\sqrt{k_{x}^{2}+k_{y}^{2}}$ is the wave-number.

The relation above rules the propagation of the gravity waves and, moreover, it defines the ω − *k* domain over which the energy of the sea waves is concentrated [22]. It is worth noting that any change of *Ū* or *h* turns into a shift of the spectral support of the sea signal. Therefore, these quantities play a key role in the analysis.

The joint estimation of the surface currents and the bathymetry performed by the NSP procedure is founded on the maximization of the following Normalized Scalar Product (Equation (2)):

(2) $$V^{j}\left(Ū,h\right)=\frac{\langle \left|F_{I}^{j}\left(\bar{k},\omega \right)\right|,G^{j}\left(\bar{k},\omega ,Ū,h\right)\rangle }{\sqrt{P_{F^{j}}\cdot P_{G^{j}}}}$$

where $\left|F_{I}^{j}\left(\bar{k},\omega \right)\right|$ is the power spectrum in the *j*th patch, *G^j^*(*k̄*, ω, *Ū, h*) is a (real) characteristic function accounting for the local support of the dispersion relation (Equation (1)), while *P_F_j* and *P_G_j* are the image power spectra $F_{I}^{j}\left(\bar{k},\omega \right)$ and *G^j^*(*k̄*, ω, *Ū, h*), respectively.

The bathymetry and the surface currents fields can thus be computed starting from the local estimates. Such information is of course extremely useful for various coastal and offshore applications, but it is also an essential tool to correctly estimate the wave field since, as it is well known, the depth and the current map are required to define a band-pass (BP) filter to separate the energy of the global sea signal from the noise background in the radar spectrum.

The required sea-wave spectrum *F_w_*(*k̄*, ω) can be obtained from the filtered image spectrum *F̃_I_*(*k̄*, ω) by resorting to the radar Modulation Transfer Function (MTF), which mitigates the distortions affecting the radar echoes and caused by both the acquisition geometry (e.g., shadowing and tilt modulation) and the electromagnetic (e.g., the Bragg) scattering mechanisms [13,22–24]. For this purpose, we use the MTF described in [22]. For the scope of this work, however, the reconstruction of the wave field is only required to establish the presence of reflected waves, as it will be shown in the following section.

## 3. Instrument and Data Description

**Figure 2.**
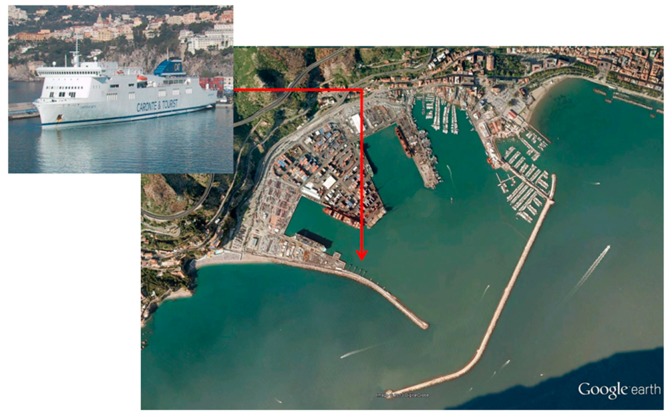
A view of Salerno Harbour and its two main jetties. The red arrow denotes the position of the acquisition system installed on the Caronte & Tourist ferry ship.

A field experiment was set up in order to assess the performances of the above described Remocean algorithm: the data were acquired by a marine radar installed on board the Caronte & Tourist ferry ship during her call in the Salerno harbour (see Figure 2) on 27 March 2013 from 11:25 a.m. to 12:00 a.m. (UTC).

Some of the characteristic parameters of the acquisition system are summarized in Table 1. In particular, the nautical radar (JRC-JMA9122-6XA) was equipped with a 6-ft antenna rotating at about 25 rpm, transmitting short pulses (70 ns) at 9.5 GHz (X-band), with a pulse repetition frequency (PRF) of 2250 Hz and an output peak power of 10 kW. Both the transmitted and the received pulses were acquired in the horizontal plane (HH polarization). The radar field of view was 360°, with an azimuth resolution of 1.2°, while the range coverage was of about 2555 m, with a range resolution of 10.5 m.

The radar system was connected to the Remocean system [22], which incorporates an analog-to-digital (AD) converter for the received signal. The radar images were stored using a 13-bit unsigned integer format, on a 1024 × 1024 pixels Cartesian grid. During the measurement campaign, the nautical radar acquired a total of 832 consecutive images, each with a pixel spacing of 5.0 m in both coordinates.

**Figure 3.**
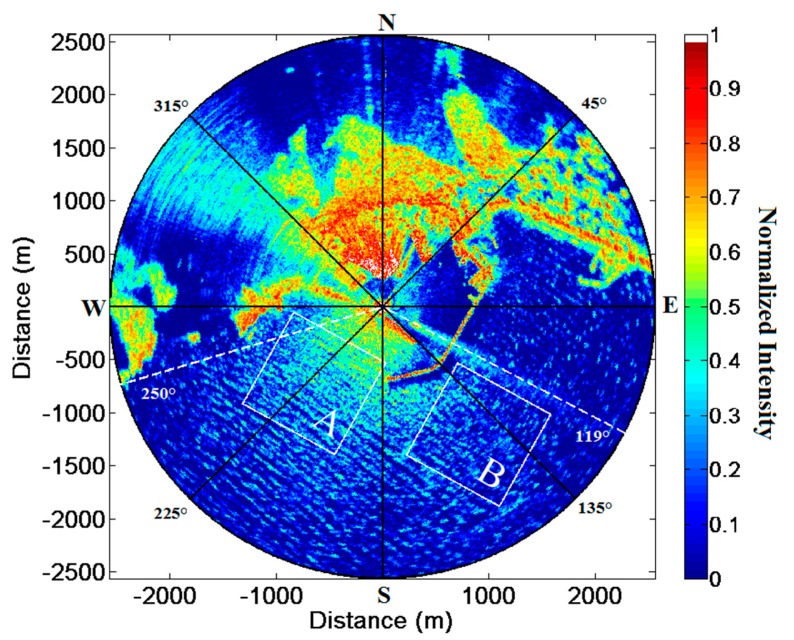
A sample image of the raw radar data set.

It is worth noting that the whole radar acquisition campaign was carried out in about half an hour, without any interference to the normal operation of the ship, thus proving the effectiveness and the flexibility of the system. A sample image of the collected dataset is shown in Figure 3. In this picture the dashed white lines define the angular sector considered for the reconstruction of the bathymetry and surface currents fields as well as for the retrieval of the sea state parameters, while the white squares identify the subareas (A and B) investigated for the detection of the sea waves reflected by the two jetties of the Salerno harbour.

## 4. Reconstruction of the Bathymetry

The reconstruction of the bathymetry field of the Salerno harbour was carried out by applying the inversion procedure described in Section 2. A sequence of 832 consecutive radar images was divided into 12 subsets, each containing 128 radar images with an overlapping factor of 64 radar images between two successive subsets. Joint current and bathymetry estimates were performed on these subsets, thus providing 12 single bathymetry maps, each with a pixel spacing of 50 m in both coordinates. A simple statistical analysis was then performed on the temporal set, so as to extract the maps of the bathymetry and of its standard deviation. The spatial resolution of each bathymetry map is dictated by the overlapping factor used in the partitioning procedure (see Section 2), which in turn derives from a trade-off between the required accuracy and the computational charge. In this work, the patch size of 500 m translated at 50 m intervals has been found to be acceptable, so the data have been analysed on a 102 × 102 grid with a spatial resolution of 50 m. A discussion of this procedure is reported in [13].

**Figure 4.**
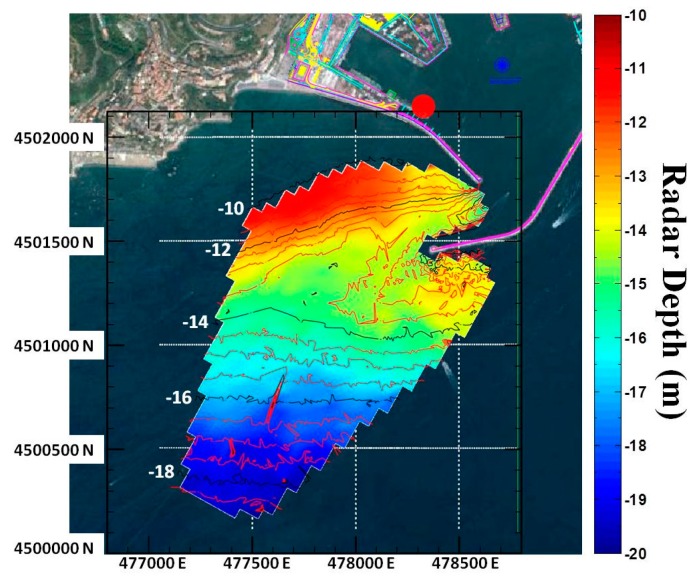
Reconstructed bathymetry map. The red circle denotes the radar location.

**Figure 5.**
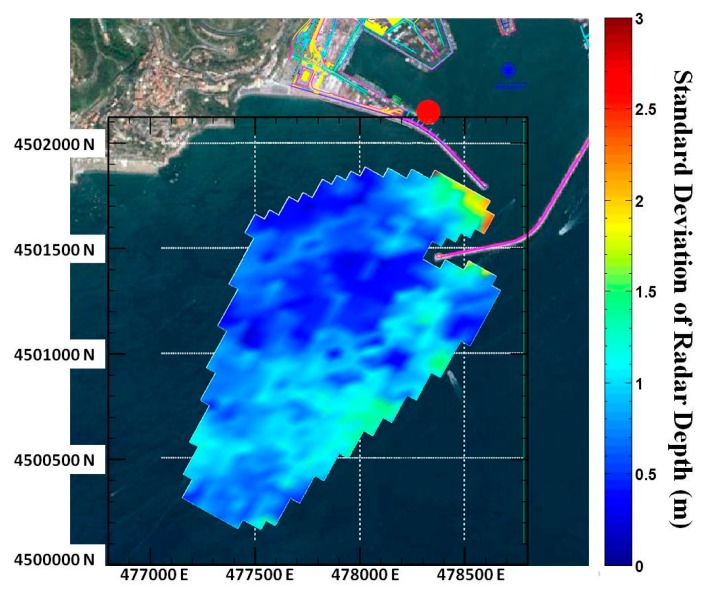
Standard deviation of radar bathymetry. The red circle denotes the radar location.

The bathymetry map is shown in Figure 4, while Figure 5 shows the local standard deviation as computed among the successive bathymetry estimates in time; its value is relatively low everywhere except in the vicinity of the seawalls and in particular at the harbour's entrance where, as expected, the low wave height and the presence of the jetties affect the accuracy of the results. Almost everywhere else the standard deviation is lower than 1 m, thus confirming the stability of the retrieval method.

**Figure 6.**
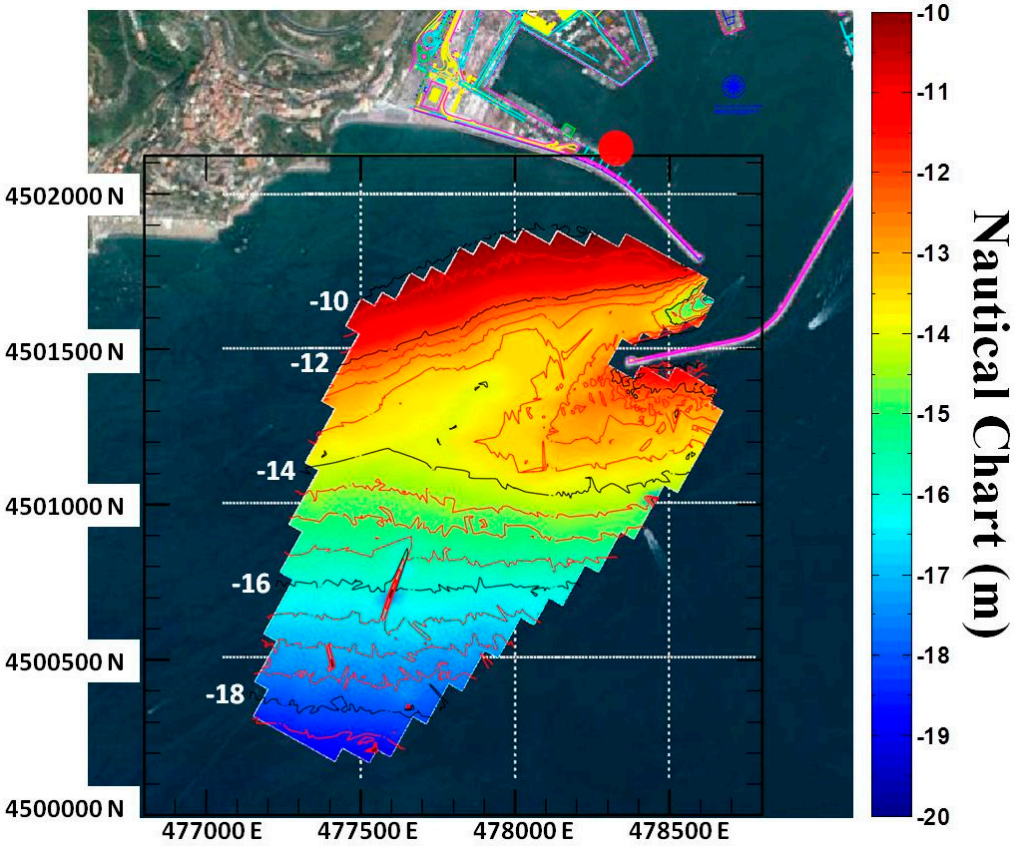
Echo-sounder bathymetry map. The red circle denotes the radar location.

The retrieved mean bathymetry field has been compared with a nautical chart obtained by a survey performed by the Port Authority, on February 2011 with a single beam ElacHydrostar 4300 echo-sounder integrated with a GPS Leica System 1200. All the measurements are referred, as usual, to the lowest values of the astronomic tide (about −0.20 m with respect to the sea mean level). The nautical chart and the radar derived bathymetry have been reported to the same grid by using a Delaunay triangulation procedure (Figure 6). In these pictures (as in Figure 4) the red and the black lines represent the iso-depth levels of the nautical chart with depth steps of 0.5 m and 2 m respectively.

**Figure 7.**
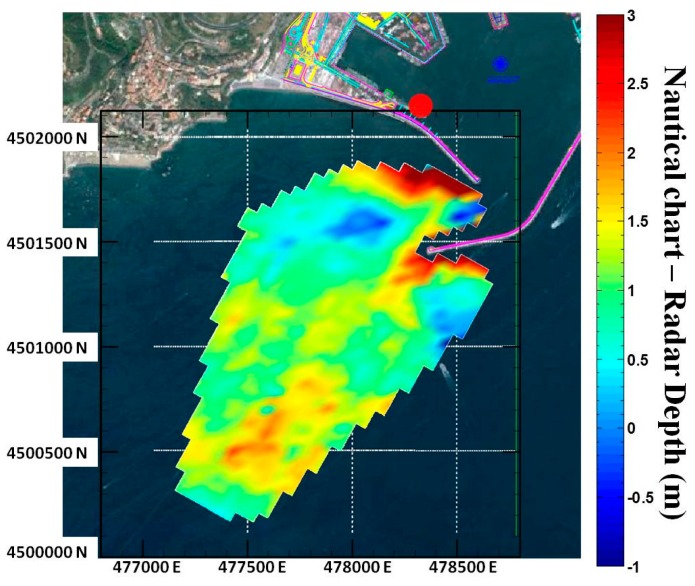
Map of differences between the echo sounder survey and the radar bathymetry.

**Figure 8.**
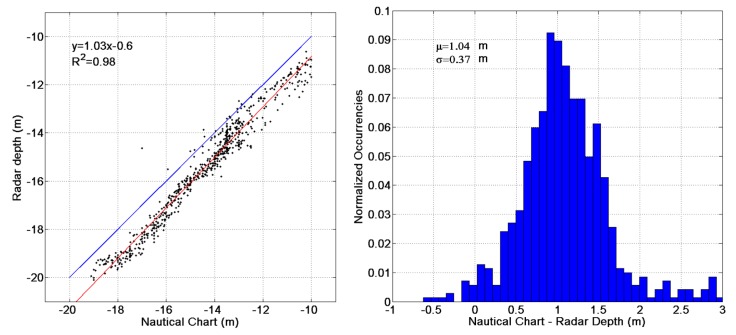
(**Left**) Radar depth *vs.* echo-sounder measurements. The red line denotes the regression line, whose equation is given in the left top side; (**Right**) Histogram of differences between the radar depth and echo-sounder measurements. The mean value (μ) and the standard deviation (σ) of the differences are given on the left top side.

The good overall reconstruction capabilities provided by the Remocean algorithm can be appreciated in Figure 7, which shows the differences between the nautical chart and the reconstructed bathymetry. As stated before, close to the jetties the error can get as high as 3 m, but as the distance increases the error reduces to less than 10% of the actual values. The performance of the system can be appreciated in the scatterplot and in the histogram shown in Figure 8.

**Figure 9.**
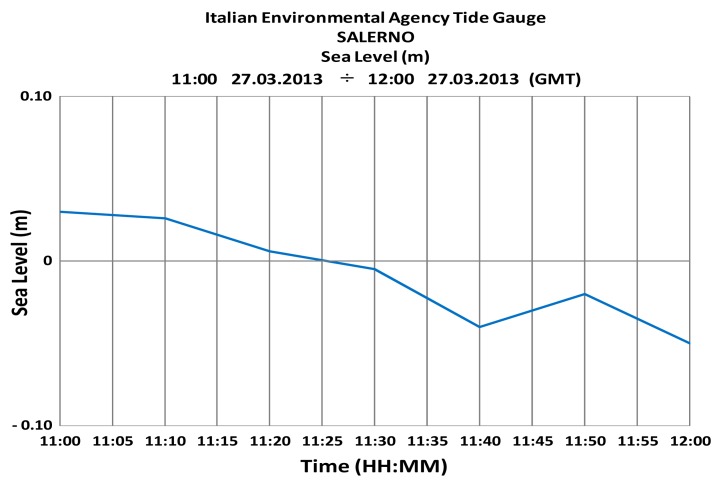
Tide level as measured by Italian Environmental Agency tide gauge during the test.

The correlation coefficient (*R*^2^ = 0.98) between the radar depth and the nautical chart is very high and the error between the radar estimates and the ground truth is very low (the standard deviation is about 0.37 m); however, a strong bias of about 1 m can be also observed. Part of this difference can be explained by the effect of tides; in order to do so, the records of the Italian Environmental Agency tide gauge, located within the harbour itself, were examined (Figure 9), and it was found that at the time of experiments the water height was at about the mean sea level (msl); since the sounding is referred to the standard datum, *i.e.*, to the average low tide level, which is about 0.20 m under the msl, at least about one fifth of the bias can be accounted for.

A further possible explanation for this systematic error can be found on the limits of the linear wave theory, upon which all the radar sea surface analyses are based. According to [16,21] the assumption of linear waves leads to an over-estimation of the water depth, and specially so in shallow water. This is indeed an aspect which needs to be clarified in further work, but the results obtained can be surely considered promising.

## 5. Sea State Retrieval and Detection of the Reflected Waves

**Figure 10.**
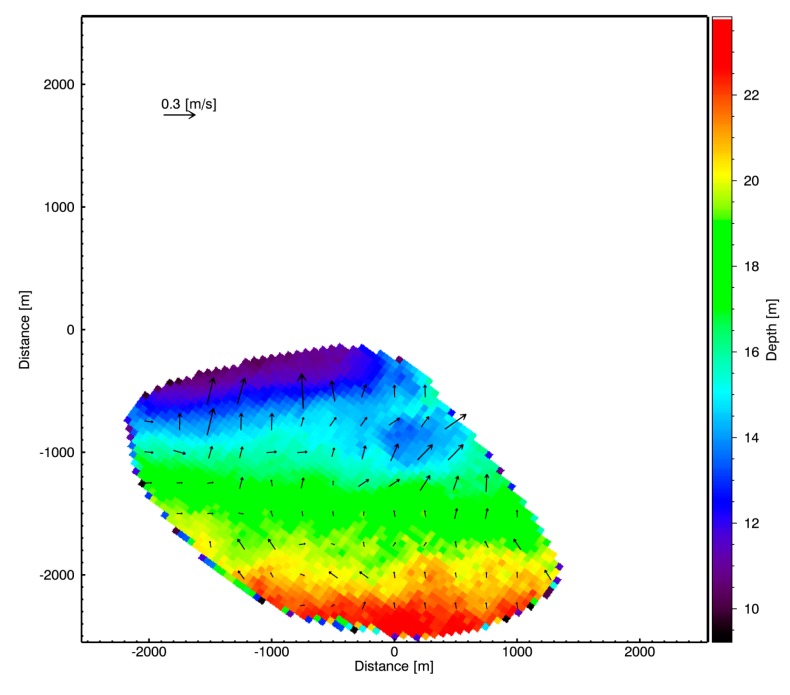
Radar bathymetry map with spatial resolution of 50 m, and the surface current vector map with a 255 m spatial resolution.

As stated earlier, the NSP method provides [7] a joint estimation procedure for both the bathymetry and the surface currents. In the particular case considered here a very low surface current field has been estimated with a mean value equal to 0.1 m/s, a maximum of about 0.3 m/s, and a standard deviation of about 0.06 m/s. No field measurements were unfortunately available. Figure 10 depicts the bathymetry map reconstructed with a spatial resolution of 50 m and an overlapping surface current vector map on a 255 × 255 grid for better visualization spatial resolution of 255 m.

The current and bathymetry values have been then employed to define the band-pass filter which is needed to compute the wave energy from the noisy radar data. Accordingly, the filtered radar spectrum *F̃_I_* (*k̄*, ω) computed for the sea region bounded by the dotted white lines in Figure 3 has been converted to the sea-wave spectrum *F_w_*(*k̄*, ω) through the Modulation Transfer Function. By integrating *F_w_*(*k̄*, ω) in the ω domain, the sea directional spectrum can be obtained and in turn the sea state parameters can be computed. The dominant wave parameters are: source direction θ*_p_* = 214° wavelength λ*_p_* = 95 m and peak period *T_p_* = 8.6 s.

No direct wave measurements were available at the experiment time, so offshore wave conditions had to be estimated by making use of European Centre for Medium-Range Weather Forecast (ECMWF, [25]), ECMWF data are produced by the “Mediterranean Model” which makes use of a Wave Action Model (WAM) coupled to the atmospheric HRES model on a 0.25° × 0.25° lat/lon grid at 6 h intervals.

**Figure 11.**
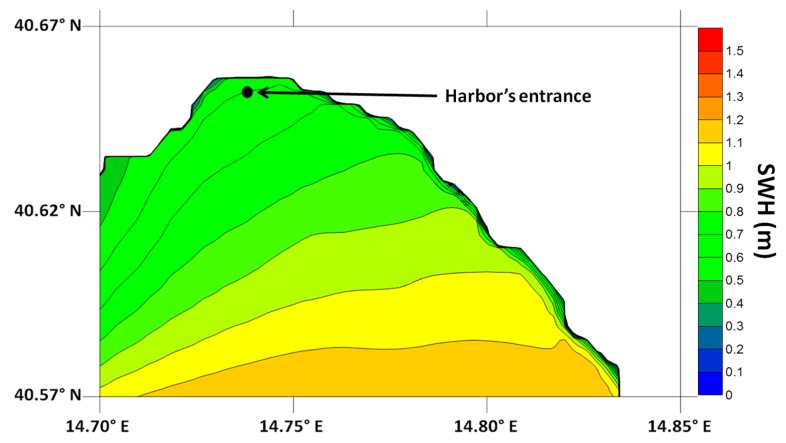
Significant Wave Height field in the Salerno bay as computed by the SWAN model.

The spatial resolution of such data is too coarse to provide an adequate understanding of the wave field in proximity of the harbour, so a local SWAN model has therefore been attached to the ECMWF grid (see Figure 11, illustrating a SWH field predicted by the Simulating Waves Nearshore (SWAN)) in order to transfer the SWH parameters from offshore to the entrance. SWAN is a widely used third generation wave model [26], developed at the Delft University of Technology [19] to compute the generation and transformation of wind-generated wave spectra in coastal areas. Its general theoretical and numerical approach is similar to many offshore wave models, such as WAM: both WAM and SWAN are driven by the predicted wind fields and use the same formulations for the source terms but, unlike the former, the SWAN model takes into account also depth induced effects, such as diffraction and wave breaking in shallow water.

Boundary conditions to the SWAN grid were those given by the ECWMF grid points closer to Salerno at 12:00 a.m. (UTC) *i.e.*, SWH 1.4 m, source direction 244°, wind speed at 10 m: 6.17 m/s, wind source direction: 220°.

Spectral balance models such as WAM and SWAN can only provide a large scale picture of the average wave field, with a resolution that is necessarily larger than the average wavelength, since neither diffraction nor reflection effects are taken into account; when a greater detail is required, specialized “near field” algorithms have to be used.

As it will be shown in the following, one of the main results of the tests which have been carried out concerns the detection of reflected waves. In order to understand and clarify the presence of such reflection, results from an elliptical Mild Slope Equation (MSE) numerical model study were applied to the test area. MSE provides a solution for a monochromatic wave field once the appropriate boundary conditions are given; the method is well known and widely used in Coastal Engineering. Equation (3) shows a typical expression for the MSE in terms of free surface elevation η(*x, y*):

(3) $$\nabla \cdot \left(a\nabla \eta \right)+{\left(1/T\right)}^{2}b\eta =0$$

where *T* is the wave period, *a* and *b* are appropriate functions of time *t* and of the local depth *h*(*x, y*), η(*x, y*) is the wave function field, which provides the local wave height. The equation above is a classical tool of Coastal Engineering [21] and the particular implementation used for this work is the Pharos Model [27], by Deltares, (formerly Delft Hydraulic Laboratories) with geometry, bathymetry and boundary conditions that had been previously used for a harbour entrance model study.

**Figure 12.**
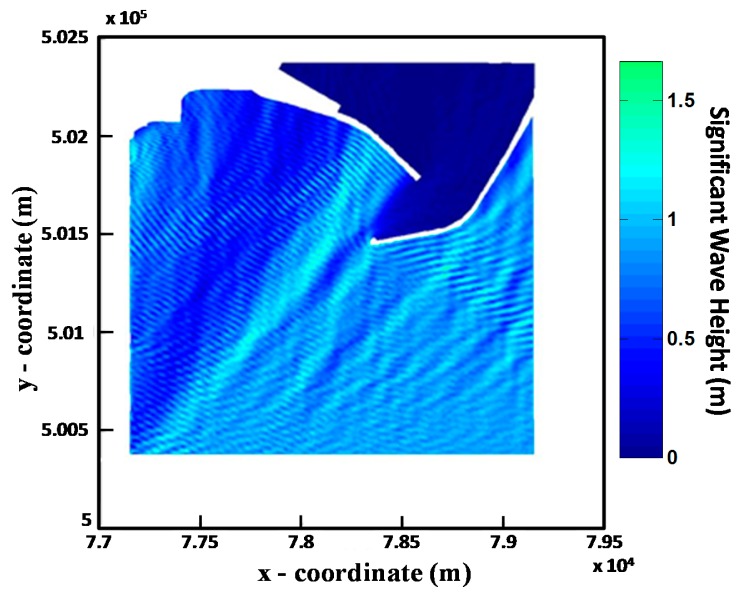
Wave field simulation of the Salerno Harbour obtained through the Mild Slope Solver.

Wave boundary conditions applied to the MSE were: SWH = 0.8 m; source direction = 209°; T = 6.3 s, *i.e.*, roughly equivalent to the mean conditions computed by the external model chain. It is important to note that this monochromatic simulation is only meant to provide an insight of the reflection phenomena outside the external breakwater at the time of the tests, and in no way it to represent an accurate picture of the wave field. Figure 12 illustrates the computed wave field accounting for reflection and diffraction phenomena occurring just outside the harbour.

As it can be seen, a whole system of reflected waves moves away from the SE facing pier and interferes with the incoming front: an effect obviously caused by reflection. A similar effect caused by the SW facing pier is also visible—less clearly since the wave propagation is nearly perpendicular to the breakwater.

**Figure 13.**
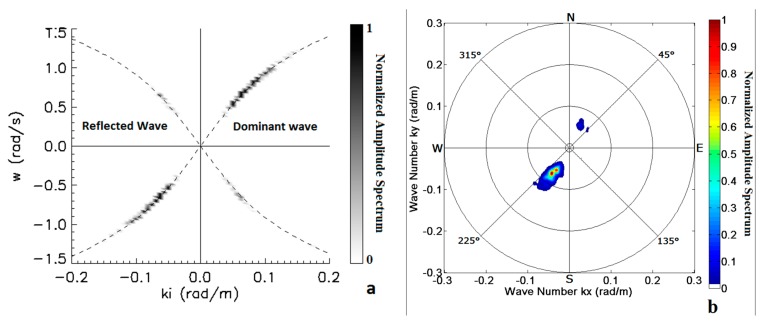
(Panel **a**) 2D section of the 3D wave spectrum relative to the subarea A. The dashed branches are representative of dispersion relation for the incident and reflected waves; (Panel **b**) 2D directional wave spectrum relative to the subarea A.

It was found that such effects can be detected by carefully analysing the radar results for two subareas A and B in Figure 3. In both cases two main spectral wave components are clearly visible, related to the incident and to the reflected wave train respectively. The source direction of the dominant wave retrieved by the Remocean system is θ*_p_* = 214°, thus impacting almost orthogonally to the SW jetty while, according to Snell's law, the reflected wave propagates along the same direction in the opposite way (*i.e.*, its direction of propagation 214°). Both these direct and reflect waves can be observed in Figure 13, which shows a 2D section along the *k̂_i_* direction (corresponding to 214°) of the 3D wave spectrum (panel a) and 2D directional spectrum (panel b) relevant to the subarea A.

In the picture the increasing (decreasing) dashed line depicts the dispersion relation for the incoming (reflected) wave train: the brighter signal represents the amplitude of the spectral density of the incident wave (coming from 214°), while the weaker signal accounts for the reflected wave (coming from 34°). In principle, the reflection coefficient could be evaluated by taking into account the relevant MTF and by considering the ratio between the spectral power of the incident and reflected wave system. In practise, as the mechanism of the radar detection of reflected is still not fully clarified, there are too many uncertainties so that only a rough estimate can be provided: for the SW breakwater its value is about 0.25, since the reflected wave contributes to the overall sea state with the 20% of the total power.

**Figure 14.**
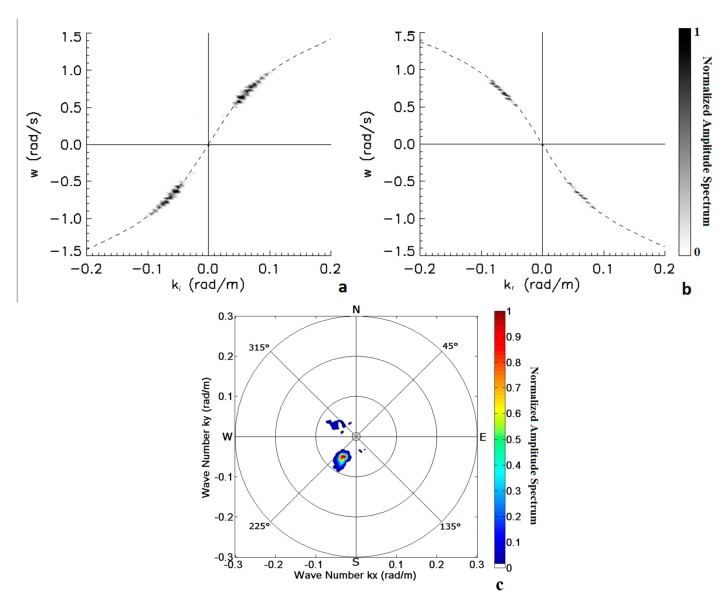
Two cuts of the 3D wave spectrum relative to the subarea B. (Panel **a**): spectral components representative of the incident wave; (Panel **b**): spectral components accounting for the reflected wave. The dashed lines represent the theoretical support of the sea waves; (Panel **c**) 2D directional wave spectrum relative to the subarea B.

As for the subarea A, the dominant wave impacts on the SE jetty of Figure 3 with an incidence angle θ*_i_* = 44° so that reflected wave propagates along a direction of about 126°; since the two wavefronts do not propagate along the same direction, they cannot be observed in a single ω − *k* cut of the 3D wave spectrum, but it is nevertheless still possible to identify the sections of the 3D wave spectrum for subarea B which contain most of the energy of the incident and reflected waves. Figure 14 shows the normalized amplitude spectrum cut along direction *k̂_i_* (left panel), which contains most of the incident wave energy, and along the direction *k̂_r_* (right panel), which contains the reflected wave. In this case we found again that the energy reflection coefficient is approximately 0.25, on the basis of the ration between reflected and total sea wave energy. These results are of course purely preliminary and incomplete, since the technique is at its initial stages.

## 6. Conclusions

A 6 ft antenna nautical X-band radar with a Remocean wave analysis system [28] has been tested at the entrance of a commercial port with a complex bathymetry. It has been shown that even in a complex scenario such a harbour entrance, a bathymetric map can be extracted by making use of a procedure which involves the spatial partitioning of the radar data and the application of the already known and tested Normalized Scalar Product (NSP) strategy. A comparison between the reconstructed bathymetry field and a recent depth survey of the area has proven that a good accuracy can be achieved as long the areas of interest are not too close to the coastline or to the piers. Even though the procedure still seems to present some bias, the result are accurate enough to provide a useful operational tool.

Besides, the bathymetry can be used to produce the Low Pass filter which is necessary to process the signal in order to derive the wave field information. It has also been shown that, once the bathymetry is estimated, an appropriate X-band radar data analysis can detect the presence of waves reflection form structures. The importance of this possibility as a support to navigation and harbour management, as well as to other coastal monitoring activities is self-evident. One of the objectives of the paper is indeed to point out that reflected waves can be detected with a navigation radar; a full theory has still to be developed, but we assume that the results are already significant at this stage. Further tests should be carried on large scale out by fitting X-band radar systems to ships in regular line services and the improvement of the reflected spectrum analysis.

## Figures and Tables

**Table 1.** Parameters of acquisition system.

**Parameter**	**Value**
Antenna rotation period (Δt)	2.4 s
Range resolution (Δr)	10.5 m
Azimuth resolution (Δφ)	1.2°
Radar scale	2555 m
Field of view	360°
Polarization	HH
Peak power	10 kW
